# Optimization of In Vitro Embryo Rescue and Development of a Kompetitive Allele-Specific PCR (KASP) Marker Related to Stenospermocarpic Seedlessness in Grape (*Vitis vinifera* L.)

**DOI:** 10.3390/ijms242417350

**Published:** 2023-12-11

**Authors:** Xiaojun Xi, Benjamin Gutierrez, Qian Zha, Xiangjing Yin, Pengpeng Sun, Aili Jiang

**Affiliations:** 1 Forestry and Pomology Research Institute, Shanghai Academy of Agricultural Sciences, Shanghai 201403, China; xiaojun_xi@saas.sh.cn (X.X.); zhaqian@saas.sh.cn (Q.Z.); yinxiangjing@saas.sh.cn (X.Y.); sunpp97@163.com (P.S.); 2 Shanghai Key Lab of Protected Horticultural Technology, Shanghai Academy of Agricultural Sciences, Shanghai 201403, China; 3 Plant Genetic Resources Unit, US Department of Agriculture-Agricultural Research Service, Geneva, NY 14456, USA; ben.gutierrez@usda.gov

**Keywords:** grape, stenospermocarpy, embryo rescue breeding, deformed seedlings, KASP

## Abstract

Seedlessness is one of the highest valued agronomic traits in grapes. Embryo rescue in combination with marker-assisted selection have been widely applied in seedless grape breeding due to the advantages of increasing the ratio of seedless progenies and shortening the breeding cycle. However, the large number of deformed seedlings produced during embryo rescue and the lack of fast, efficient, and low-cost markers severely inhibit the process of seedless grape breeding. In this study, a total of eighty-three grape cultivars (51 seedless and 32 seeded) with diverse genetic backgrounds and two populations derived from embryo rescue, including 113 F1 hybrid individuals (60 seedless and 53 seeded), were utilized. We screened suitable media for converting malformed seedlings into normal seedlings, analyzed the association between the SNP in *VviAGL11* and seeded/seedless phenotype, and developed a KASP marker related to stenospermocarpic seedlessness. Our results indicated that the transformation rate of 37.8% was obtained with MS medium supplemented with 2.0 mg·L^−1^ of 6-BA and 0.5 mg·L^−1^ of IBA. The presence of an A nucleotide allele at position chr18:26889437 was further confirmed to be fully associated with the stenospermocarpic seedlessness phenotype. The developed KASP marker, based on the verified SNP locus in *VviAGL11*, successfully distinguished the seedless and seeded genotypes with high precision and throughput. The results will contribute to enhancing the efficiency of embryo rescue and facilitate parent selection and early selection of seedless offspring with molecular markers, thereby accelerating the breeding process in seedless table grapes.

## 1. Introduction

Grapes (*Vitis* species) are highly significant fruit crops worldwide, which have been utilized for centuries for direct consumption (table grapes), as a source of juice and wines (wine grapes), or as dried grapes with an extended shelf life (raisin grapes) [1]. The seedlessness trait is highly valued in table grapes, making it a significant global trend in grape production and consumption as well as a key objective in grape breeding [2]. Seedless grapes are botanically grouped into parthenocarpic or stenospermocarpic; the former does not rely on pollination, while the latter involves embryo abortion and subsequent regression in seed development after full fertilization [3]. In parthenocarpy, fruits grow without fertilization, producing small berries, and the process is associated with impaired meiosis. Therefore, the most commercially important seedless grape varieties are stenospermocarpic due to their berries being larger than in parthenocarpic grapes. Stenospermocarpy is heritable, but the proportion of seedless progenies has been reported to be less than 15% when inherited from the paternal parent [4]. In vitro culture prevents immature embryo death in the stenospermocarpic berries by initially plating fertilized ovules on a growth medium to allow embryo growth beyond the stage of abortion, and then, by opportunely culturing the newly developed embryos until germination and plantlet formation. Embryo rescue from stenospermocarpic maternal parents greatly increases the ratio of seedless progenies and shortens the breeding cycle [5,6]. Furthermore, various aspects have been explored to optimize embryo rescue and enhance its efficiency, including parental selection, sampling timing, media composition, culture techniques, and the application of plant growth regulators [7,8,9]. Injury of the embryo during excision or unsuitable growth conditions can result in high proportions of deformed seedlings and limit population sizes. It has been reported that a medium supplemented with plant growth regulators permitting the development of malformed seedlings into plantlets facilitates the production of more progenies, though further optimization is needed [9,10].

Marker assisted selection (MAS), which could enhance the efficiency of seedless trait selection by shortening breeding duration and optimizing the breeding process during the protracted juvenile phase, is essential for seedless grape breeding due to the expensive and time/space-consuming nature of conventional breeding [11]. Previous studies have provided evidence that *VviAGL11*, which consists of eight exons spanning ~7.6 kb with a coding region of 672 bp (NCBI: KM401845 cv. Chardonnay) and is located on linkage group (LG) 18, plays a causal role in stenospermocarpic grapevines [12,13,14]. The expression of *VviAGL11* in pea-sized berries (when seeds begin to develop) was 25 times higher in the seeded homozygous genotype compared to the flower stage in the seedless homozygous grape genotype [12]. Several molecular markers for assessing seedlessness have been developed and widely extended to grape varieties and hybrids with diverse genetic backgrounds [12,15,16,17,18,19]. Recently, a strong correlation was observed between the missense mutation Arg197Leu in exon 7 of *VviAGL11* and seedlessness in grapevines [20]. However, as a result of the limited availability of seedless grape cultivars, only 20 out of the 124 grape cultivars selected in Royo’s study were seedless. Therefore, the availability and practicality of this SNP in *VviAGL11* needs to be further verified in larger population.

KASP (kompetitive allele-specific PCR), a high-throughput, flexible, low-cost, and intelligent genotyping method, has extensive applications in the field of agriculture, animal sciences, and human health. Compared to other genotyping techniques, KASP is based on fluorescent signals and does not require the laborious gel electrophoresis system. Thus, the technology has been extensively applied in fine mapping, marker assisted selection, and rapid cultivar identification in grape [21,22,23] and other plants, such as maize [24], soybean [25], and wheat [26].

Embryo rescue coupled with MAS could serve as a powerful tool to overcome various obstacles in the process of seedless grape breeding, including poor breeding efficiency, high costs, and lengthy breeding cycles. However, the production of a significant number of deformed seedlings during embryo rescue and the lack of fast, efficient, and cost-effective markers remain major challenges in breeding seedless grapes. This work aimed to optimize embryo rescue protocols by screening suitable media for converting malformed seedlings into normal seedlings. Moreover, the association between the SNP in *VviAGL11* and seeded/seedless phenotypes was analysis and verified. A KASP molecular marker was developed and validated to provide fast, efficient, and low-cost markers for seedless grape breeding. The results will contribute to enhancing the efficiency of embryo rescue and supply technical support for parent selection and early selection of seedless hybrid offspring with molecular markers, thereby accelerating the process of seedless grape breeding.

## 2. Results

### 2.1. Impact of Various Plant Growth Regulators on Developing Deformed Seedlings into Plantlets

**Figure 1:**
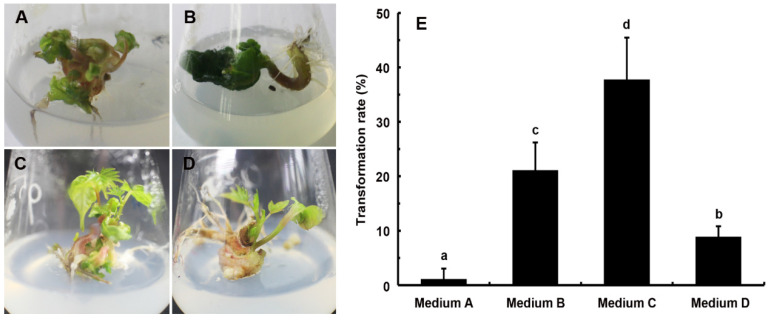
Transformation and utilization of the deformed seedlings. (**A**,**B**) Deformed seedlings. (**C**,**D**) Deformed seedlings grown into normal plantlets. (**E**) Transformation rate of different media. Data are mean ± SD (*n* = 3). The lowercase letters indicate statistically significant differences with *p* < 0.05.

The process of embryo rescue often results in the occurrence of malformed seedlings, which are characterized by abnormal cotyledons or roots, and sometimes even the absence of cotyledons or roots (Figure 1A,B). The transformation rate of deformed seedlings into normal plantlets varied significantly between media with different growth regulator concentrations (Figure 1E). The malformed seedlings inoculated on medium A (without plant growth regulators) could barely convert into normal plantlets with only a 1.1% transformation rate. The transformation rates of deformed seedlings cultured on medium B, medium C, and medium D were 21.1%, 37.8%, and 8.9%, respectively. The medium containing a combination of 6-benzylaminopurine (6-BA) and indole-3-butyric acid (IBA) exhibited the highest transformation rate, which was higher than those with 6-BA or IBA separately.

### 2.2. Validation of SNP in VviAGL11 Related to Stenospermocarpic Seedlessness

We randomly selected thirty-two seeded grapevines used as food, wine, and rootstock, which belong to *V. vinifera*, hybrids of *V. vinifera-labrusca*, *V. berlandieri-riparia*, *V. labrusca-riparia*, and *V. berlandieri-rupestris*, respectively. All of these grape cultivars presented hard seeds with totally sclerified integuments in ripe berries during two consecutive years. Each seedless grapevine was also found to be absent of seeds or with seed traces present in mature fruits during 2019–2020. According to the PCR–Sanger sequencing data of a collection of 83 accessions, the C/C genotype at position chr18:26889437 was fully associated with the seeded phenotype (Table 1). In the stenospermocarpic seedless grapevine germplasm population, each individual displayed the A/C genotype at this SNP locus with 100% coincidence rate. Interestingly, the seedless phenotype attributed to parthenocarpy or meiotic abnormalities, such as ‘Concord Seedless’, ‘Niagara Seedless’, and ‘Summer Black’, had no association with the A/C genotype at chr18:26889437, but presented the C/C genotype at this locus (Table 1).

According to two years of seed phenotype assessment, forty-eight and forty-three progenies in the cross between ‘Centennial Seedless’ and ‘Shine Muscat’ were absent and present for seeds, respectively. Consistent with the results in the natural germplasm population, the C/C and A/C genotype at position chr18:26889437 in CS × SM were completely related to the 43 seeded and 48 seedless phenotypes, respectively (Table 2). The chi-square test showed that both the genotype at this locus and the phenotype were in accordance with the expected Mendel segregation ratio of 1:1 (χ^2^_c_ = 0.2747 < χ^2^_0.05_ = 3.841).

A total of twenty-two progenies were obtained from the cross between ‘Centennial Seedless’ and ‘Himrod’, including twelve seedless and ten seeded seedlings, respectively. Similarly, the seeded and seedless phenotype in this cross combination were fully associated with the C/C and A/C genotype at chr18:26889437, respectively (Table 2). Interestingly, we observed three seedless offspring with the A/A genotype at this locus. Therefore, the presence of the A nucleotide allele at chr18:26889437 was deemed to be in connection with stenospermocarpy. Based on the chi-square test, the genotype at this locus in CS × HM was segregated in compliance with the Mendelian 1:2:1 ratio (χ^2^_c_ = 5.1818, *p* < 0.05), while seedlessness was not consistent with an expected 3:1 phenotype ratio (χ^2^_c_ = 4.9118, *p* > 0.05).

### 2.3. Development and Validation of KASP Marker Related to Stenospermocarpy

**Figure 2:**
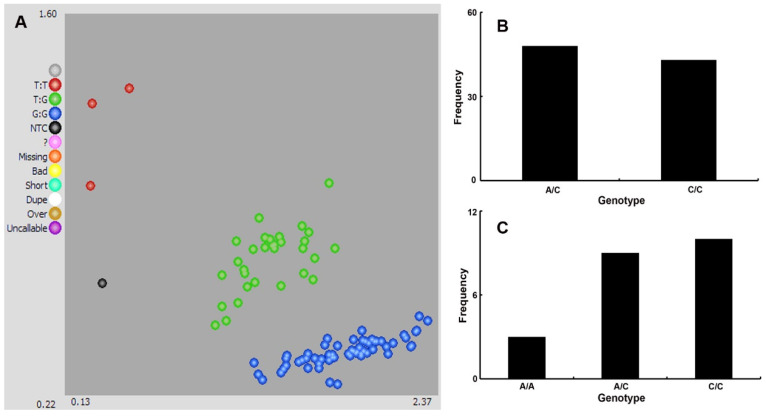
KASP-labeled fluorescence detection results and segregation within the progeny population. (**A**) Genotyping results using the KASP assay technique. The red and blue dots denote the homozygous alleles and the green dots denote the heterozygous alleles. (**B**) KASP genotyping in CS × SM. (**C**) KASP genotyping in CS × HM.

To make full use of the above-verified SNP in *VviAGL11*, we further developed a KASP marker and validated it in a total of 83 grapevine germplasm samples and 113 F1 population individuals. Three genotypes were displayed in the KASP marker with allele Y (red) and heterozygous type (green), related to the seedless trait, and allele X (blue), associated with the seeded phenotype, respectively (Figure 2A). The genotypes detected with KASP in the natural population were consistent with those detected with PCR–Sanger sequencing. Likewise, the KASP genotyping in two cross populations was in accordance with phenotyping and sequencing data (Figure 2B,C).

## 3. Discussion

Since Ramming and Emershad [27] initially acquired their new seedless grape cultivars through embryo rescue from seedless female parents, this technique has been extensively employed by plant breeders to rescue inherently weak, immature, and/or abortive embryos as well as to overcome the failure of endosperm development in interspecific, intergeneric, and interploid hybridizations [28,29,30]. Due to the large proportion of deformed seedlings obtained during embryo rescue, the efficiency of seedless grape breeding through embryo rescue is severely restricted [9]. Malformed seedlings without normal development, even if temporarily viable under laboratory conditions, would eventually die after transplantation from defects in morphology and function. Therefore, it is crucial to enhance the efficiency of embryo rescue to increase breeding population sizes. Media containing suitable concentrations and combinations of plant growth regulators have contributed to the transformation of malformed seedlings to normal plantlets [31]. It has been reported that the WPS medium modified with 5.0 mg·L^–1^ of IBA and 2.0 mg·L^–1^ of 6-BA transformed malformed seedlings to develop them into plantlets with a rate of 40.91% in ‘Flame Seedless’ [9]. The addition of 2.0 mg·L^−1^ of 6-BA and 2.0 mg·L^−1^ of IAA to the WPM medium has proven to be suitable for normalizing the aberrant seedlings in ‘Thompson Seedless’ and ‘Heshi Seedless’ [10]. In the present study, the MS medium supplemented with 2.0 mg·L^−1^ of 6-BA and 0.5 mg·L^−1^ of IBA was found to achieve the highest transformation rate of 37.8% in ‘Centennial Seedless’.

Recently, the role of *VviAGL11* as the primary factor responsible for seedlessness in grape cultivars has been established, and a causative mutation in CDS has been identified [20]. The substitution of Arg-197Leu in *VviAGL11* has been suggested to prevent the activation of gene expression, then hinder seed coat differentiation, and ultimately lead to endosperm degeneration and embryo developmental arrest in seed traces. In this study, the presence of the A nucleotide allele at position chr18:26889437 was further confirmed to be fully associated with the stenospermocarpic seedlessness phenotype. However, we indicated that this SNP is ineffective in the parthenocarpic seedless cultivars, which attributed to the fundamentally distinct mechanism underlying seedlessness formation against stenospermocarpy. Similarly, the formation of seedlessness in triploid grapevines due to meiotic abnormalities was irrelevant to the mutation in *VviAGL11*. Three seedless progenies resulting from the cross between ‘Centennial Seedless’ and ‘Himrod’ displayed a homozygous A/A genotype at this locus, resembling that observed in a seedless × seedless cross [20], which could serve as a potential parent for breeding of seedless grapes. Previous reports suggested that the seedless rate of progenies obtained through embryo rescue (16.7–92%) greatly increased compared to that via conventional crossbreeding (0–49%) [32]. In the present study, the seedless rates in F1 individuals obtained from CS × SM and CS × HM were 52.7% and 54.5%, respectively, which was basically in accordance with previous reports in seedless × seeded or seedless × seedless crosses [33,34]. The seedless rates in F1 individuals were suggested to be greatly affected by cross combination and the different seedless heritability of the parental genotype, especially the maternal genotype [8]. Therefore, parental varieties with high heritability of the seedless trait should be selected for seedless grape breeding to enhance the seedless rates in progenies. Moreover, the seedless trait segregated in the seedless × seeded cross in this study was basically consistent with the expected 1:1 ratio, which was also reported in previous research [35,36,37]. The inconsistent segregation of seedless trait in the seedless × seedless cross might be attributed to limited number of progenies obtained in this study. However, more studies indicated that the segregation of seedlessness in F1 hybrids was not in accordance with the Mendelian ratio [7,34,38]. Thus, stenospermocarpic seedlessness is a complex trait and both the genetic and molecular bases of seedlessness need to be further investigated in the future.

In grapevine, MAS has been widely applied to screen individuals with favorable alleles for seedlessness at the early phase, which could advance the breeding efficiency with decrease in the costs, space, and time for varietal development and phenotypic evaluation. A series of molecular markers related to seedlessness were developed and utilized in grape germplasm and hybrid populations with diverse genetic backgrounds; among them SSR markers p3_VvAGL11, VMC7f2, and 5U_VviAGL11 were reported to exhibit enhanced accuracy and efficiency [37,38,39]. However, SSR genotyping based on PCR and amplicon size analysis by electrophoresis (polyacrylamide gel electrophoresis or capillary electrophoresis) were still not simple, fast, and low-cost enough to achieve assisted selection purposes. KASP is a high-throughput, flexible, and economical genotyping platform, which could detect SNPs with high precision and has a potential application in MAS. In the present research, we developed a KASP marker based on the verified SNP locus in *VviAGL11* associated with stenospermocarpic seedlessness and explored its applicability and accuracy in a grapevine germplasm and hybrid population derived from a stenospermocarpic parent. Altogether, this KASP marker could distinguish the seedless and seeded genotypes with high precision and throughput in accordance with phenotypic evaluations and sequencing data. The applicability and accuracy of this designed KASP related to stenospermocarpic seedlessness should be further measured in other hybrid populations with different genetic backgrounds, thus improving its application in seedless grape breeding.

Grafting promotes early fruit production and ensures genetic consistency in numerous economically significant fruit tree crops. The scions of *Vitis vinifera* are commonly grafted onto various rootstocks of other *Vitis* species to influence scion vigor, modulate fruit composition and provide resistance to biotic and abiotic stresses [40]. Despite these advantages, different combinations of rootstock and scion have been found to exhibit variations in their phenotypic effects [41,42]. Additional factors contributed to phenotypic variation include the age of the grafted individuals, genotype × environment and rootstock genotype × scion genotype × environment interactions [43,44]. To the best of our knowledge, there have been no reports regarding the influence of grafting, age, and environmental factors on the seedless trait in grapes. However, the complex interactions between the rootstock, cultivar, and environment necessitate controlling for genotypic/age variation/environment in further research to ensure that the effects of genotype/age/eco-physiology on the success of identifying seedless fruits are not overlooked. The utilization of machine learning (ML) has been reported to enhance the accuracy of predictions in a tree-breeding context, specifically in terms of “plus tree” selection and hybrid breeding by the integration of environmental variables, microhabitat diversity, and genome-wide divergence, as well as the adaptation to climate change in natural forests [44]. Therefore, these “big data” ML approaches, capable of comprehensively integrating heterogeneous genomic and ecological datasets, are strongly encouraged for further investigations into seedlessness in grapes.

Apart from grapes, seedlessness is also a valuable agricultural trait in other fruit crops, such as citrus, persimmon, and banana, which enhances the fruit’s eating quality by increasing the amount of edible pulp and eliminating the presence of hard seeds with an unpleasant taste [45,46]. The technique of embryo rescue has recently gained widespread application in breeding programs aimed at achieving seedlessness, as it allows for the successful recovery of non-viable embryos that would otherwise perish during traditional plant breeding practices. Moreover, precise and efficient MAS for the seedless trait during the seedling stage is crucial to enhancing breeding efficiency and minimizing the associated costs. The alteration of multiple *AGL11* homologs (MADS-box gene members of class D) has been linked to seed initiation/development and the program for setting seedless fruit, as supported by several lines of evidence [47,48]. Thus, the embryo rescue-based seedlessness breeding approach, combined with the MAS developed in this study, could potentially be utilized as part of horticultural tree improvement programs, as well as at nurseries by following these steps: (1) cultivate hybrid seedlings via embryo rescue technology until they reach the stage of 5–7 true leaves; (2) identify seedless germplasms with the genotypes of A:A or A:C using the KASP marker; (3) transplant the finally selected seedless germplasms to the field for management. Moreover, the propagation of cultivars from most tree fruit species is traditionally challenging when using their own roots, regardless of whether layering or cutting techniques are employed [49]. The yield and fruit quality were found to be influenced by various propagation methods as well as the eco-physiological context [50]. Therefore, the investigation of clonal propagation performance for seedlessness traits across different propagation methods and environmental conditions is highly deserving of future research.

## 4. Materials and Methods

### 4.1. Plant Materials

Twenty-four seedless grape cultivars were maintained in the USDA-ARS cold-hardy grape collection (Geneva, NY, USA). Twenty-seven seedless grape cultivars and thirty-two randomly selected seeded grapevines were cultivated in an experimental vineyard at the Shanghai Academy of Agricultural Sciences (Fengxian District, Shanghai, China). The characteristics of those germplasm resources were listed in Table S1. Two hybrid populations listed in Table S2 were cultivated in an experimental vineyard at the Shanghai Academy of Agricultural Sciences (Fengxian District, Shanghai, China). The first F1 population was derived from a cross between *Vitis vinifera* cv. Centennial Seedless (CS) and *V. vinifera* × *V. labrusca* cv. Shine Muscat (SM). The second population was derived from a cross between *V. vinifera* cv. Centennial Seedless (CS) and *V. vinifera* × *V. labrusca* cv. Himrod (HM). The selected cultivars were mature (aged 6–30 years) and in good health, exhibiting a bountiful harvest. The daily management of grapevines involves ensuring adequate irrigation, nutrient supply, and pest management. Young leaves of the natural population and F1 hybrids were sampled, rapidly frozen in liquid nitrogen, and subsequently stored at a temperature of −80 °C for further analysis.

### 4.2. Embryo Rescue

**Figure 3:**
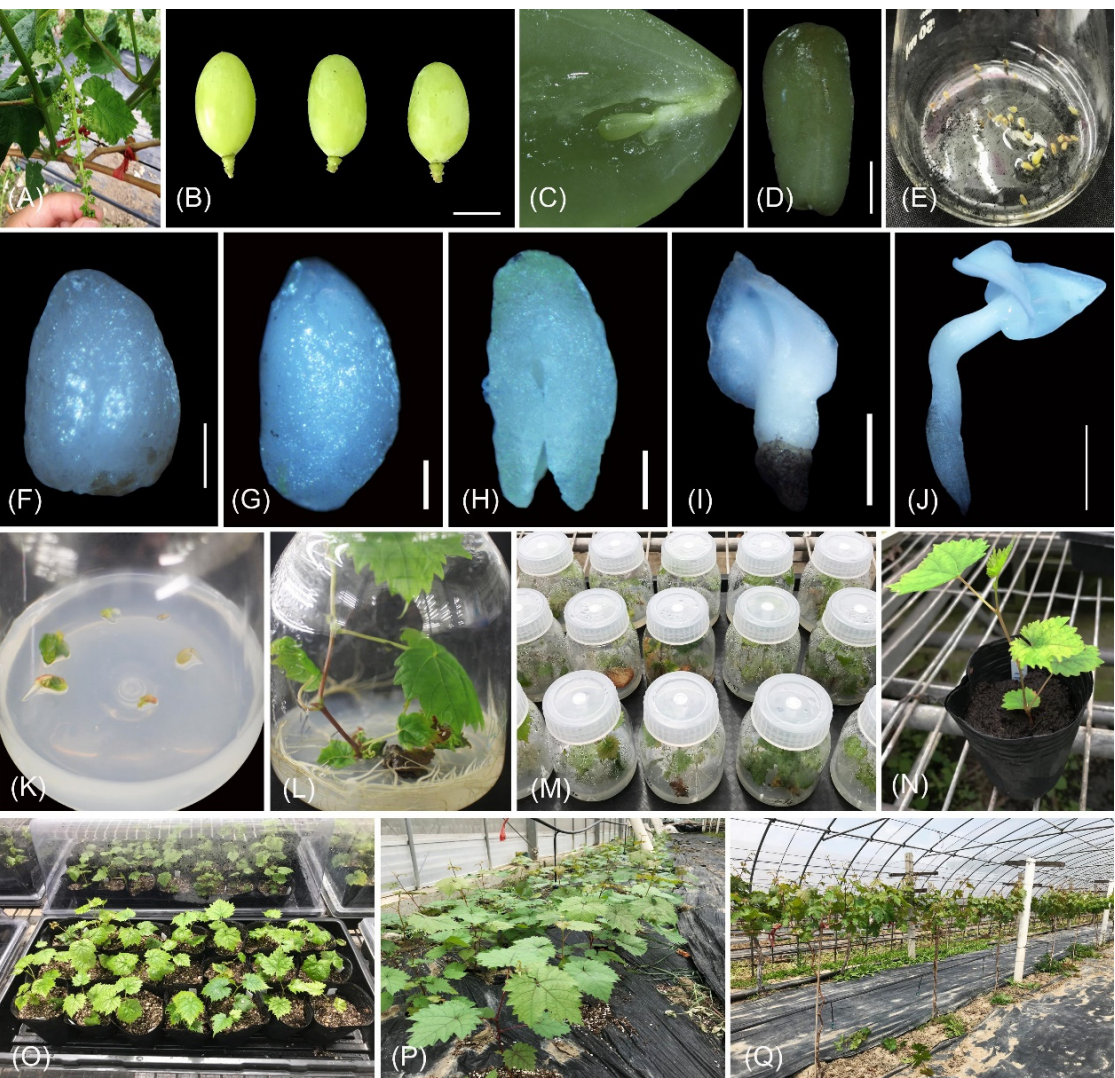
The process of in vitro embryo rescue technology. (**A**) Artificial emasculation of ‘Centennial Seedless’. (**B**) Immature hybrid fruits collected at 30 DAP (scale bar = 1 cm). (**C**) Dissected berry with ovule. (**D**) Isolated ovule (scale bar = 1 mm). (**E**) Ovules cultured in liquid medium. (**F**) Globular embryo (scale bar = 200 µm). (**G**) Heart-shaped embryo (scale bar = 200 µm). (**H**) Torpedo-shaped embryo (scale bar = 100 µm). (**I**,**J**) Cotyledon-shaped embryo (scale bar = 1000 µm and 2000 µm, respectively). (**K**) Immature embryo germination. (**L**) Plantlet from subculture. (**M**) Seedlings acclimatization. (**N**) Seedlings transported to nutrition bowls. (**O**) Seedlings cultured in greenhouse. (**P**) Seedlings transported in field. (**Q**) Seedlings growing in vineyard.

‘Shine Muscat’ and ‘Himrod’ pollen was collected and stored in a bottle containing silica gel at 4 °C, respectively. Artificial emasculation and hybridization were performed on ‘Centennial Seedless’ in May at the Shanghai Academy of Agricultural Sciences (Figure 3A). Embryo rescue was performed according to the method established in our lab with minor modification [51]. Briefly, immature hybrid fruits were sampled 30 d after pollination (DAP) and subjected to a thorough rinsing under running water for a duration of 2–3 h (Figure 3B). The following steps were conducted under a sterile condition, wherein berries were immersed in 70% alcohol for 30 s, subsequently rinsed twice with sterile water, agitated in a 0.5% sodium hypochlorite solution for 20 min, and finally rinsed three times with sterile water. After sterilization, the ovules were isolated and placed into 50 mL glass flasks with 3 mL liquid culture medium (Figure 3C–E). The flasks were each evenly distributed with approximately 20 ovules, which contained 1/2 MS medium supplement with 30 g∙L^–1^ sucrose and 1.0 g∙L^–1^ activated charcoal (AC), and the pH was adjusted to 5.8. The glass flasks were incubated in a dark environment for 14 weeks at 25 ± 1 °C. The developed embryos were carefully dissected from the ovules using a scalpel under a stereoscope and transferred onto MS supplemented with 0.2 mg∙L^–1^ 6-BA, 6.0 g∙L^–1^ agar, and 30 g∙L^–1^ sucrose (Figure 3F–K). The embryos were cultivated at a temperature of 25 ± 1 °C, with exposure to white fluorescent light (40 μmol∙m^–2^∙s^–1^) for 16 h each day. The plantlets were propagated with the subculture media of 1/2 MS supplemented with 0.2 mg∙L^–1^ IBA, 6.0 g∙L^–1^ agar, and 20 g∙L^–1^ sucrose (Figure 3L). After 2 months, the residual medium surrounding the roots was meticulously rinsed away, and the plantlets were subsequently transplanted into nutrition bowls containing a synthetic soil mixture composed of peat, perlite, and vermiculite in a ratio of 3:1:1 (*v*/*v*/*v*) once they reached a larger and more robust size (Figure 3M,N). About twenty plantlets were placed on a plate covered with a transparent plastic film to retain warmth and humidity, and cultured in greenhouse (Figure 3O). After 2–3 weeks, the plastic film was removed and the plantlets were allowed to grow for a duration of one month prior to being transplanted into the field (Figure 3P). Finally, the surviving seedlings were planted in an experimental vineyard (Figure 3Q).

### 4.3. Screening Medium for Deformed Seedlings into Plantlets

The cross between ‘Centennial Seedless’ and ‘Shine Muscat’ was employed to identify an appropriate culture media capable of enhancing the growth of malformed seedlings into viable plantlets. Malformed seedlings were cultivated on MS media supplemented with various plant growth regulators as specified in Table 3. After 4 weeks, the number of malformed seedlings into plantlets was counted. Percent transformation rate = Number of plantlets/Number of seedlings malformed ×100.

### 4.4. Seed Phenotype Assessment

During 2019–2020, seed phenotype was assessed with 20 randomly sampled berries from each individual at harvest. Based on the previous report, the presence of individuals with hard seeds characterized by completely sclerified integuments was defined as seeded individuals. The presence of soft seed traces with unsclerified or partially sclerified integuments and absence of seeds were regarded as seedless individuals [3].

### 4.5. Validation of SNP in VviAGL11

The genomic DNA was extracted using an HP Plant DNA Kit (Omega) following the manufacturer’s instructions. After checked the quality and quantity with NanoDrop 2000 spectrophotometer (Thermo Fisher Scientific, Waltham, MA, USA), the DNA samples were diluted to 50 ng·μL^−1^ and subsequently stored at −20 °C for further PCR analysis. The verification of SNPs was performed using PCR-based Sanger sequencing. Briefly, the PCR reactions using the PrimeSTAR HS DNAPolymerase (Takara, Dalian, China) were performed in a 25 μL volume containing 5 μL of 5× Buffer (Mg^2+^ Plus), 2 μL of dNTP Mixture (2.5 mM), 0.25 μL of Polymerase (2.5 U/μL), 1 μL of each primer (10 μM), 1 μL of gDNA, and 14.75 μL of ddH_2_O. Details of the primer information is listed in Table S3. The amplification process was initiated by a heat activation step at 95 °C for 10 min, followed by 35 cycles consisting of denaturation at 94 °C for 1 min, annealing at 60 °C for 1 min, extension at 72 °C for 1 min, and a final extension step at 72 °C for 7 min. The PCR products were subjected to Sanger sequencing using sequence-specific primers, and the resulting sequences were compared and analyzed by Sequencher software (version 4.9, Gene Codes Corporation, Ann Arbor, MI, USA).

### 4.6. Development and Validation of the KASP Marker

The genotyping analysis was performed using the high-throughput PCR SNPline system (LGC, Biosearch Technologies, Teddington, UK), following the protocol provided in the KASP manual. For this SNP locus, three pairs of primers including two competitive allele-specific forward primers and one common reverse primer were designed and listed in Table S4. PCR amplification was conducted according to the previous report [22]. At the end of the amplification, the plates were subjected to fluorescent reading, and SNPviewer 2.0 software (LGC, Biosearch Technologies, Teddington, UK) was utilized for allele calling.

### 4.7. Statistical Analyses

Data were presented as the average of three independent replicates. Significance was analyzed using one-way ANOVA (*p* < 0.05) in SPSS 18.0 (SPSS Inc., Chicago, IL, USA), and differences were compared using Duncan’s multiple range test, respectively.

## 5. Conclusions

The medium consisting of MS supplemented with 2.0 mg·L^−1^ of 6-BA and 0.5 mg·L^−1^ of IBA was identified as the optimal culture medium for promoting the conversion of malformed seedlings into normal plantlets, achieving a transformation rate of 37.8%. The stenospermocarpic seedlessness phenotype was completely related to the presence of A nucleotide allele at position chr18:26889437. According to the verified SNP locus in *VviAGL11*, a low-cost, high-throughput KASP marker was developed and validated to distinguish the seedless and seeded individuals. Therefore, the combination of embryo rescue and MAS in this study is expected to enhance and expedite the breeding process for seedless grape cultivars.

## Figures and Tables

**Table 1:** Verification of seeded or seedless grape germplasm by PCR–Sanger sequencing analysis.

	Phenotype		No.	Genotype	Cultivar	No.	Coincidence Rate (%)
Naturalpopulation	Seeded		32	C/C	Shine Muscat, Muscat of Alexandria, Zaoheibao, Graca, Xinya, Shennong Jinhuanghou, Guiyuan, Yan 73, Alicante Bouschet, Brazil, Ruidu Hongmei, Muscat Hamburg, Rosario Bianco, Rizamat, Tamina, Ruidu Xiangyu, Victoria, Ruidu Kemei, 3E-16-23, Manicure Finger, Jingxiangyu, Moldova, Shen’ai, G-26, SO4, Beta, 1103P, Benni Fuji, Pione, 16–32, 16–33, 13–30	32	100
	Seedless	Stenospermocarpy	44	A/C	SP275, Golerura, Centennial Seedless, RuiduWuheyi, Bronx Seedless, Black Seedless, ZhengyanWuhe, Blush Seedless, Aishen Meigui, Superior Seedless, Yuehong Seedless, Thompson Seedless, Hongyan Wuhe, Himrod, Venus Seedless, 98-38, G-8, 98-1, 98-2, 21-60, 99-212, 96-89, 20-A-2, G-18, Green Seedless-1, Green Seedless-2, Canadice, Bronx Seedless, Sovereign Coronation, Vanessa Seedless, Hendrickson Seedless, Stout Seedless, Einset Seedless, Mars, Reliance, Gervan, Remaily Seedless, III 39-1, Himrod 4×, Challenger, Lakemont, Romulus, Suffolk Red, Interlaken	44	100
		Parthenocarpy or meiotic abnormalities in triploids	7	C/C	Summer Black, Zaoxiahei, Niagara Seedless, Concord Seedless, Ruifeng Seedless, Royal Seedless, Christmas A	7	0

**Table 2:** Verification of seeded or seedless hybrids by PCR–Sanger sequencing analysis.

	Phenotype	No.	Genotype	Hybrid	No.	Coincidence Rate (%)
CS × SM	Seeded	43	C/C	CS1, CS3, CS4, CS8, CS12, CS15, CS16, CS17, CS18, CS21, CS22, CS23, CS24, CS27, CS30, CS31, CS32, CS36, CS37, CS41, CS44, CS49, CS53, CS54, CS56, CS58, CS59, CS61, CS63, CS64, CS65, CS67, CS69, CS70, CS75, CS79, CS80, CS84, CS85, CS86, CS87, CS89, CS90	43	100
	Seedless	48	A/C	CS2, CS5, CS6, CS7, CS9, CS10, CS11, CS13, CS14, CS19, CS20, CS25, CS26, CS28, CS29, CS33, CS34, CS35, CS38, CS39, CS40, CS42, CS43, CS45, CS46, CS47, CS48, CS50, CS51, CS52, CS55, CS57, CS60, CS62, CS66, CS68, CS71, CS72, CS73, CS74, CS76, CS77, CS78, CS81, CS82, CS83, CS88, CS91	48	100
CS × HM	Seeded	10	C	CH1, CH4, CH5, CH6, CH7, CH8, CH10, CH17, CH18, CH21	10	100
	Seedless	9	A/C	CH2, CH12, CH13, CH14, CH15, CH16, CH19, CH20, CH22	9	100
		3	A/A	CH3, CH9, CH11	3	100

**Table 3:** The content of the four media.

Medium	Composition
Medium A	MS + 30 g∙L^–1^ sucrose + 6 g∙L^–1^ agar
Medium B	MS + 2.0 mg∙L^–1^ 6-BA + 30 g∙L^–1^ sucrose + 6 g∙L^–1^ agar
Medium C	MS + 2.0 mg∙L^–1^ 6-BA + 0.5 mg∙L^–1^ IBA + 30 g∙L^–1^ sucrose + 6 g∙L^–1^ agar
Medium D	MS + 2.0 mg∙L^–1^ IBA + 30 g∙L^–1^ sucrose + 6 g∙L^–1^ agar

## Data Availability

Data is contained within the article and supplementary material.

## Supplementary Materials

The supporting information can be downloaded at: https://www.mdpi.com/article/10.3390/ijms242417350/s1.
